# A Microplate-Based Approach to Map Interactions between TDP-43 and α-Synuclein

**DOI:** 10.3390/jcm11030573

**Published:** 2022-01-24

**Authors:** Angelo M. Jamerlan, Seong Soo A. An

**Affiliations:** Department of Bionano Technology, Gachon Medical Research Institute, Gachon University, Seongnam-si 13120, Korea; angelojamerlan@gmail.com

**Keywords:** epitope mapping, TDP-43, alpha synuclein, ELISA, comorbidity, proteinopathy, aggregation

## Abstract

Trans-active response DNA-binding protein (TDP-43) is a multifunctional regulatory protein, whose abnormal deposition in neurons was linked to debilitating neurodegenerative diseases, such as amyotrophic lateral sclerosis, frontotemporal lobar degeneration, Limbic-predominant age-related TDP-43 encephalopathy, and Alzheimer’s disease with a secondary pathology. Several reports showed that TDP-43 proteinopathy as a comorbidity can form aggregates with other pathological proteins. The co-deposition of alpha synuclein and TDP-43 inclusions was previously reported in glial cells and by observing TDP-43 proteinopathy in Lewy body disease. In this study, it was hypothesized that alpha synuclein and TDP-43 may co-aggregate, resulting in comorbid synucleinopathy and TDP-43 proteinopathy. A solid-phase microplate-based immunoassay was used to map out the epitopes of anti-TDP-43 antibodies and locate the interaction of TDP-43 with α-synuclein. A region of the low complexity domain of TDP-43 (aa 311–314) was shown to interact with full-length α-synuclein. Conversely, full-length TDP-43 was shown to bind to the non-amyloid beta component of α-synuclein. Using in silico sequence-based prediction, the affinity and dissociation constant of full-length TDP-43 and α-synuclein were calculated to be −10.83 kcal/mol and 1.13 × 10^−8^, respectively. Taken together, this microplate-based method is convenient, economical, and rapid in locating antibody epitopes as well as interaction sites of two proteins.

## 1. Introduction

Trans-active response DNA-binding protein (43 kDa) (TDP-43) is a regulatory protein linked to various roles in transcriptional repression, mRNA splicing, and regulation of protein translation. It was discovered as an HIV-1 expression regulatory element that functions by binding to trans-active response (TAR) elements [1]. TDP-43 has four main domains, starting from the N-terminus: a nuclear localization signal (NLS), two RNA-recognition motifs (RRMs), and a low-complexity C-terminal domain (LCD) [2,3,4]. Structural mutations in the C-terminal region of TDP-43 cause abnormal localization and deposition of the protein within neuronal cytoplasm, resulting in diseases such as amyotrophic lateral sclerosis (ALS), and frontotemporal lobar degeneration (FTLD) [4,5]. TDP-43 pathology in these diseases is widely varied and affects different cell types and brain regions. This heterogeneity may be a result of genetic and environmental risk factors, giving rise to varied distribution patterns of the underlying pathology in specific brain regions as the disease progresses [6]. TDP-43 was reported to co-localize with other protein species characteristic in other neurogenerative diseases, namely Huntington’s disease, Parkinson’s disease (PD), dementia with Lewy bodies (DLB), and Alzheimer’s disease (AD) [7,8,9,10,11]. Interestingly, TDP-43 isoforms were observed in brain tissue of neuropathologically confirmed AD cases using different antibodies that target full-length or are restricted to the C-terminal regions of TDP-43 [12].

Alpha synuclein is a 14.4 kDa presynaptic neuronal protein commonly associated with PD and DLB. The protofibrillar species of α-synuclein is commonly considered toxic and leads to disruption of cellular homeostasis by interfering with several intracellular targets as well as synaptic function [13]. Propagation of synucleinopathy is then aggravated by seeding and accumulation of proteinaceous aggregates. Alpha synuclein has three main domains: N-terminal lipid binding α-helix, non-amyloid-binding central domain (NAC), and C-terminal acidic tail [14]. The NAC is hydrophobic and promotes aggregation [15,16,17]. Interestingly, TDP-43 pathology was identified through immunohistochemical analysis in 31% of DLB with AD cases, 7.2% in PD cases, and 19% on PD cases with dementia (PDD), while none were found in DLB and one case in normal controls [8]. Hence, the possible interaction of TDP-43 and α-synuclein warranted further investigation.

In this study, a solid-phase microplate-based approach was used to locate epitopes of anti-TDP-43 antibodies, and to characterize the location of interactions of TDP-43 with full length recombinant alpha synuclein. This method was used similarly to map affinity interactions of prion with amyloid-beta (Aβ)-42, and to investigate interactions between myofilament proteins [18,19]. Here, epitopes of different anti-TDP-43 antibodies were identified, as well as the sites of interaction of recombinant TDP-43 with full length recombinant alpha synuclein, and the sites of interaction of recombinant alpha synuclein with full length recombinant TDP-43.

## 2. Materials and Methods

### 2.1. Peptide Synthesis and Preparation

**Figure 1:**
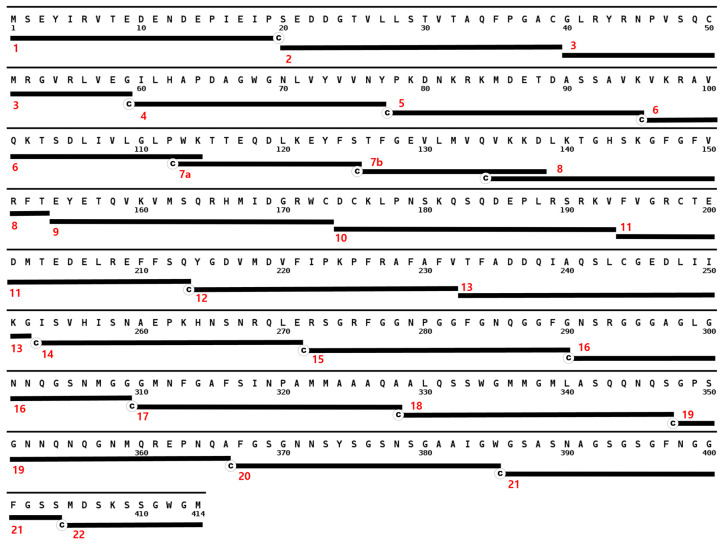
Synthesized TDP-43 peptides aligned with the complete sequence of TDP-43. The sequence starts with the N-terminus at the upper left corner of the figure and ends with the 414th residue at the bottom. Peptides are represented by black bars with cysteine tags and are numbered at the left.

**Figure 2:**
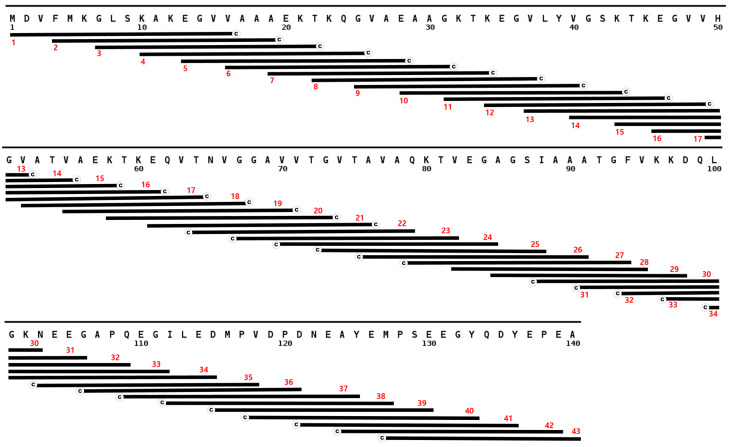
Overlapping α-synuclein peptides aligned with the complete sequence of α-synuclein. The sequence starts with the N-terminus at the upper left corner of the figure and ends with the 140th residue at the lower right corner. Peptides are represented by black bars with cysteine tags and are numbered at the left.

Twenty-three non-overlapping peptides that cover the complete amino acid sequence of TDP-43 (Figure 1), and 43 overlapping peptides that cover alpha synuclein (Figure 2) were synthesized (Lugen Sci Co., Ltd., Bucheon, Korea). Each peptide has a length of 18 to 20 amino acids, except 2 that cover the first few residues of RRM-1 (13-mers) and the last residues of the LCD (10-mer) in TDP-43. A terminal cysteine was added to all peptides without an internal cysteine residue. These lyophilized peptides were stored in an airtight container inside a dehumidifier at room temperature until use. In preparing the peptides to be coated on to a maleimide-activated microplate (ThermoScientific^®^, Waltham, MA, USA), 0.5 to 1.0 mg peptide were dissolved in an appropriate volume of dimethyl sulfoxide (DMSO, Amresco, Dallas, TX, USA) to make a stock solution of 10 mg/mL. These peptides were then diluted to a final concentration of 5 µg/mL in phosphate buffer saline (PBS) solution with 10 mM EDTA. One hundred µL of each peptide solution was then transferred to a corresponding well of a maleimide-activated plate (ThermoScientific^®^, Waltham, MA, USA), and incubated overnight at 4 °C.

### 2.2. Microplate-Based Epitope Mapping of Antibodies and Recombinant Proteins

**Figure 3:**
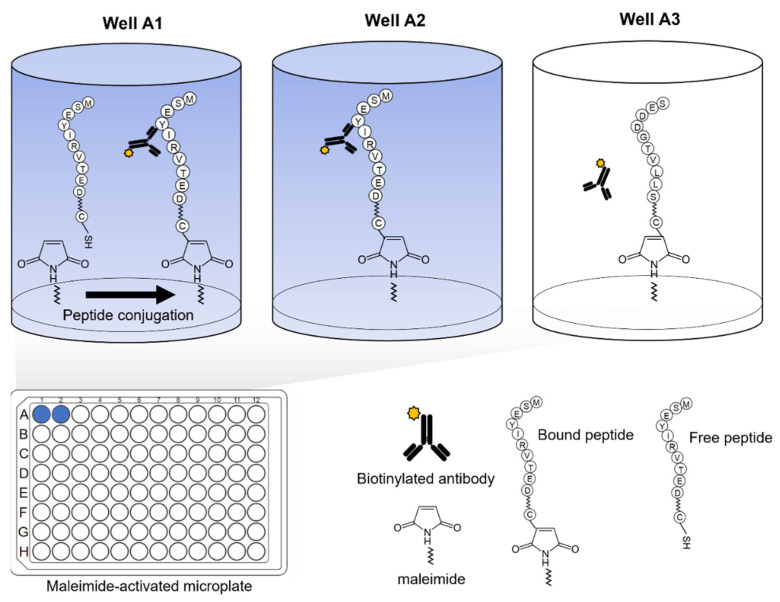
Microplate-based epitope mapping using maleimide-coated plates. Peptide sequences are coated separately on the well surfaces and bind through covalent interaction of cysteine with maleimide. Protein interactions are detected through a visible colorimetric reaction on the well.

After washing all coated wells three times with PBS with 0.05% Tween-20 (PBST), 100 µL of 10 µg/mL of L-cysteine (Sigma, Burlington, MA, USA) dissolved in PBS was added to all wells and left to incubate at room temperature for 1 h to block remaining exposed maleimide groups. After washing three times with PBST, 100 µL of 0.1 µg/mL of interacting recombinant protein to be tested was added next and left to incubate for 1 h at 37 °C. In mapping the epitopes of the antibodies for each peptide, no recombinant protein was added, and the antibodies were directly added instead on to the coated wells (Figure 3). After washing, 100 µL of 0.1 µg/mL of the biotinylated detection antibody diluted in PBST (10% BlockAce; Bio-Rad, Hercules, CA, USA) was added and left to incubate for 1 h at 37 °C. After washing, 100 µL of streptavidin conjugated with horseradish peroxidase (ThermoScientific, Waltham, MA, USA) was diluted 5000 times in PBST with 10% BlockAce and left to incubate for 30 min at room temperature. After a final washing, 100 µL of tetramethylbenzidine (TMB; ThermoScientific) was added and left to incubate at room temperature until a significant color change relative to the background was observed (around 15 to 20 min). The reaction was stopped using 50 µL of 1 M sulfuric acid, and absorbance values were measured at 450 nm using a PerkinElmer Victor 3 multilabel reader.

### 2.3. Interaction of α-Synuclein and TDP-43

Three µg/mL of α-synuclein and TDP-43 diluted in PBS were coated on separate polystyrene plates (ThermoFisher, Waltham, MA, USA), and incubated overnight at 4 °C. The plates were then washed three times with PBST and blocked with 200 µL of 3% bovine serum albumin (BSA; Millipore, Burlington, MA, USA) diluted in PBS for 1 h at room temperature. After washing, 100 µL of serially diluted (50, 10, and 2 ng/mL) of recombinant TDP-43 and α-synuclein in PBST were applied on to α-synuclein and TDP-43 coated wells, respectively. The plates were then left to incubate for 1 h at 37 °C. After washing, 100 µL of 0.1 µg/mL of biotinylated TDP-43 antibodies (10782-2-AP and 12892-1-AP; Abcam, Cambridge, UK) diluted in PBST with 10% BlockAce, and anti-α-synuclein antibodies (FL-140; Santa Cruz Biotechnology, Dallas, TX, USA) were applied on wells treated with TDP-43 and α-synuclein, respectively, and left to incubate for 1 h at 37 °C. After washing, the wells were treated with 100 µL of HRP-conjugated streptavidin diluted 10,000 times in PBST with 10% BlockAce and left to incubate for 30 min at room temperature. After washing, chemiluminescent substrate (SuperSignal ELISA Pico, Thermo Scientific) was added to achieve greater sensitivity. The resulting luminescence was then measured using a Victor 3 multilabel reader.

### 2.4. In Silico Sequence-Based Prediction

ISLAND is an online program that incorporates machine learning to calculate the affinity and dissociation constant (k*_d_*) of two protein sequences. The sequences of aa 310–329 (and full length α-synuclein were entered into the program, and their affinity and k*_d_* were calculated.

### 2.5. Statistical Analysis

Assays were performed in triplicate and an independent t-test was used to evaluate any statistical significance between the generated absorbances and the background absorbance (JASP, University of Amsterdam, Amsterdam, The Netherlands). The differences were considered significant at *p* < 0.05.

## 3. Results and Discussion

### 3.1. Epitope Mapping of Anti-TDP-43 Antibodies

**Figure 4:**
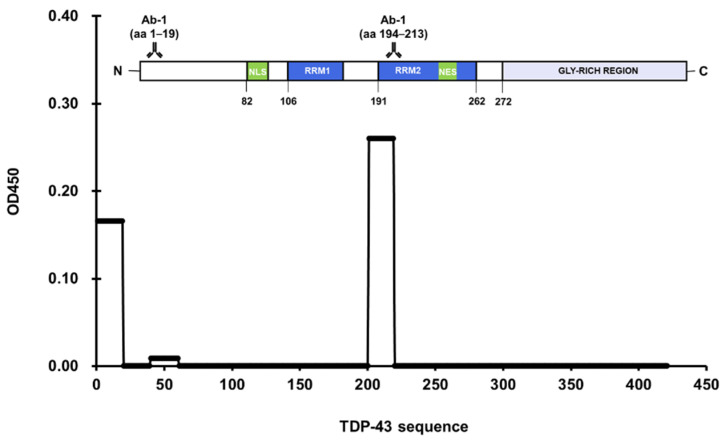
Significant absorbance values were found in regions aa 1–19 and aa 194–213 for polyclonal anti-TDP-43 antibody (10782-2-AP).

**Figure 5:**
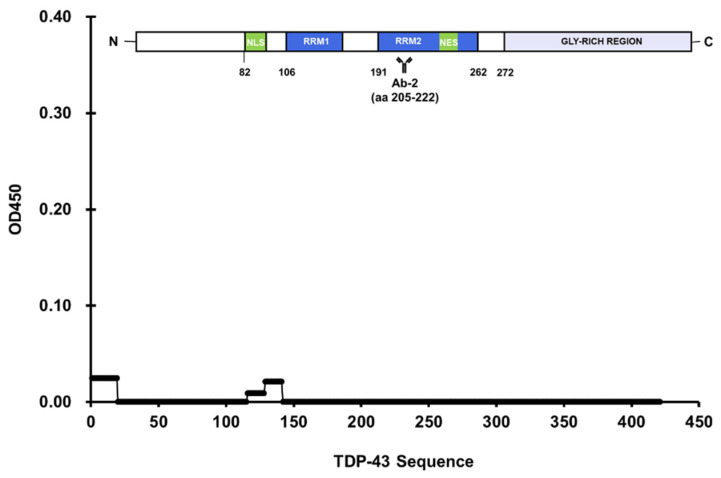
No significant reaction in all TDP-43 peptides was observed during treatment with Ab-2 (2E2D3).

**Figure 6:**
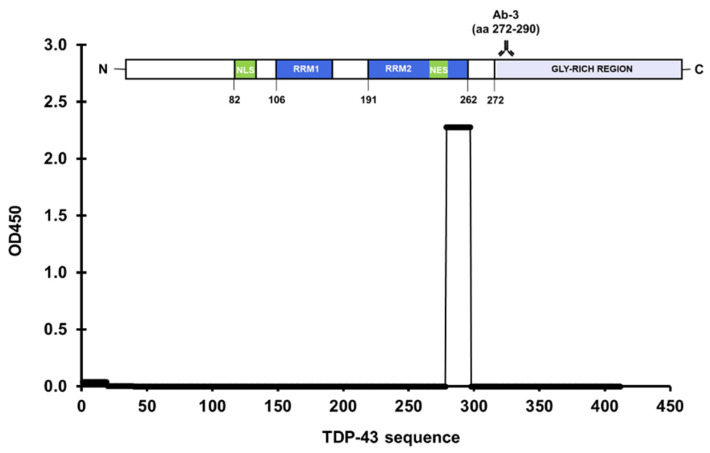
A significant absorbance was observed in aa 272–290 for polyclonal anti-TDP-43 antibody (12892-1-AP).

Three antibodies were selected from a systematic literature review of 671 anti-TDP-43 antibodies featured in publications from 2006 to 2014 [20]. The list of publications was condensed by excluding ones that utilized non-human subjects or did not directly apply anti-TDP-43 antibodies such as in genetics studies. Further refining the list and removing duplicates amounted to 38 unique antibodies, Ab-1 (10782-2-AP) ranked first in the 10 highest-ranking antibodies based on positive scores resulting from immunostaining or Western blot assays [20]. Results of the microplate-based approach showed two peptides with a positive reaction: peptide 1 (aa 1–19), and peptide 11 (aa 194–213) (Figure 4). Peptide 1 belonged to the NTD, and peptide 11 belonged to RRM-2. This antibody was documented to have two epitopes, aa 203–209, and a residue near the NTD [21]. Ab-2 (2E2-D3; Abnova, Taipei, Taiwan) ranked second and binds to aa 205–222 [22]. However, no identifiable reaction resulted in all peptides treated with this antibody (Figure 5). TDP-43 residues 205–222 were fragmented between peptides 11 and 12, which consisted of aa 194–213 and aa 214–232, respectively. This demonstrated the limitation of the approach in this study if the epitope happens to be fragmented across multiple peptides. Finally, Ab-3 (12892-1-AP) was ranked fifth in the list [20]. A significant reaction was observed in the well coated with peptide 15, aa 272–290 (Figure 6). Still, Ab-3 was selected for this study since it was generated from protein immunogens and was likely to be non-specific or influenced by epitope variations. In contrast, the other antibodies that ranked third and fourth were also specific for the LCD but were generated from peptide immunogens and were not as specific.

### 3.2. Full Length Alpha Synuclein Interacts with a Region of the LCD in TDP-43

**Figure 7:**
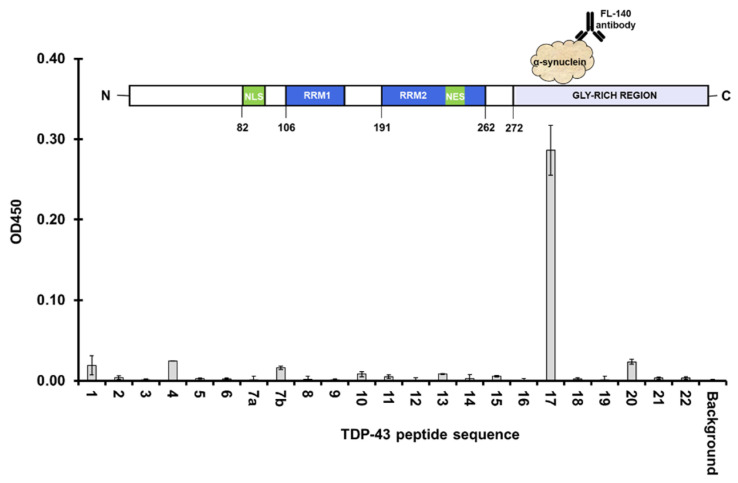
Probing epitope interactions of TDP-43 with other recombinant proteins using a microplate-based approach. Peptide 17 (aa 310–329) of TDP-43 showed a significant amount of bound full length α-synuclein.

**Figure 8:**
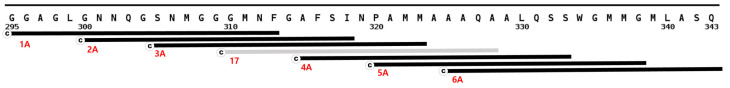
Design of overlapping TDP-43 peptides relative to the region of interest (aa 310–329). Only peptides 1A, 3A, and 4A were successfully synthesized.

Using the same microplate-based approach, the TDP-43 peptides were separately treated with three different recombinant proteins: albumin, tau, and α-synuclein. Results showed that only full length α-synuclein produced a significant reaction in the well coated with peptide 17 (aa 310–329), a region in the LCD of TDP-43 (Figure 7). To better resolve the residues that contributed most to the interaction within this region, overlapping peptides that shift every five residues and span aa 291–347 (peptides 16–18) were designed (Lugen Sci Co., Ltd., Bucheon, Korea). However, only three out of six peptides were successfully synthesized. The remaining peptides were then treated with full length α-synuclein, and only aa 295–313 and aa 305–323, as well as aa 310–329 showed a significant binding reaction (Figure S1). MNFG (aa 311–324) residues were shared between these regions, and likely contributed to the interactions with full length α-synuclein (Figure 8).

### 3.3. Full Length TDP-43 Interacts with the NAC Region of α-Synuclein

**Figure 9:**
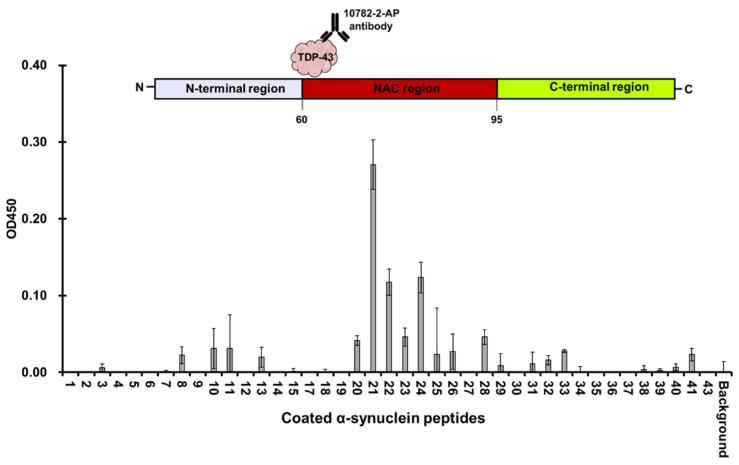
Full length TDP-43 bound most significantly to the first 16 residues of the NAC domain of α-synuclein.

To investigate the region in α-synuclein which full length TDP-43 interacts with, 43 overlapping peptides of α-synuclein that shift every three residues were coated on to the wells and treated with full length TDP-43. Amino acids 61–76 of α-synuclein showed the highest reaction with TDP-43 (Figure 9). Interestingly, this sequence marked the first 16 residues of the hydrophobic non-amyloid β component (NAC) of α-synuclein. The NAC region was known to induce aggregation in synucleinopathies. This result showed that the same region was not limited to induce self-aggregation but may interact with other proteins with high affinity like TDP-43 within its proximity. The co-localization of TDP-43 and α-synuclein was reported in glial cytoplasmic inclusions in paraffin-embedded tissue sections of the amygdala and basal forebrain of autopsy-confirmed multiple system atrophy (MSA) cases using double stain immunohistochemistry and immunogold electron microscopy [23]. This supported the likelihood that the two proteins could interact. TDP-43 pathology was also reported in Lewy body diseases, like PD, but were not co-localized with α-synuclein staining. However, this rare co-localization of the two proteins was still observed in dystrophic neurites [8,24]. Recently, the interaction of the LCD of TDP-43 and full-length α-synuclein was characterized through nuclear magnetic resonance (NMR), and chemical shifts were more prominent in the N and C-terminal ends of α-synuclein after incubation with TDP-43 LCD [25]. The authors stated that TDP-43 LCD was likely to interact electrostatically in these regions since these were hydrophilic and acidic. Binding to TDP-43 LCD can then gather the NAC regions of α-synuclein with enough proximity for aggregation to occur [25]. This was interesting, since the peptide containing the first 15 residues of the NAC region of α-synuclein showed the highest absorbance for bound full-length TDP-43 (Figure 9). Perhaps the difference resulted from shorter segments (15-mer) of α-synuclein coated on separate wells and that these were incubated with full-length TDP-43. The shorter peptides from the N and C-termini of α-synuclein may not have had enough residues to produce a sufficient affinity towards full length TDP-43, but there may have been enough hydrophobic residues in the peptides within the NAC region to bind to TDP-43. In contrast, the NMR result of Dhakal et al. was acquired from the incubation of full-length α-synuclein and TDP-43 LCD and it can be surmised that this increased the likelihood for TDP-43 to preferably interact with the hydrophilic regions of α-synuclein. In addition, Dhakal et al. mentioned a loss in cross-peaks in the NAC region due to aggregation [25]. In our approach, a region of the NAC was isolated as a peptide, coated, and incubated with full-length TDP-43. Bound TDP-43 was then quantified using Ab-1. Thus, a significant interaction of full length TDP-43 was identified in the first 15 residues of the NAC region of α-synuclein. It seemed then that the NAC region of α-synuclein also had an essential role in TDP-43 interaction apart from aggregation.

### 3.4. Three-Dimensional Scatterplots of the Peptides Reveal Similar Properties That Influence Their Interaction

**Figure 10:**
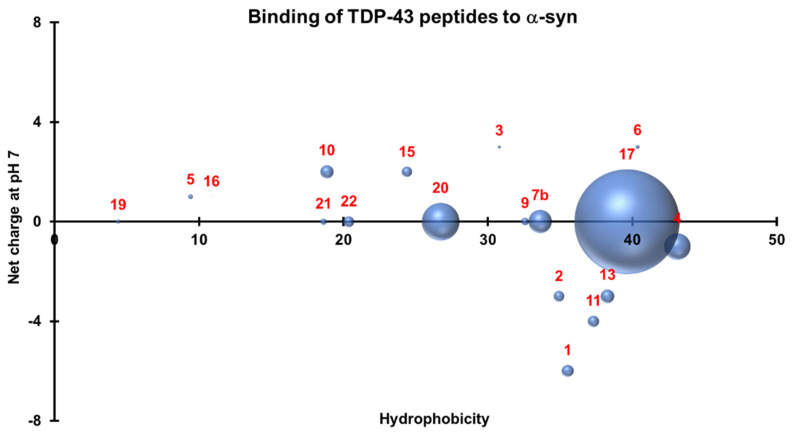
Three-dimensional scatterplot of TDP-43 peptides based on their hydrophobicity indices, net charge at pH 7.0, and their interactions with α-synuclein. Significant interactions occur with neutral peptides with hydrophobicity indices that fall within 20–40.

**Figure 11:**
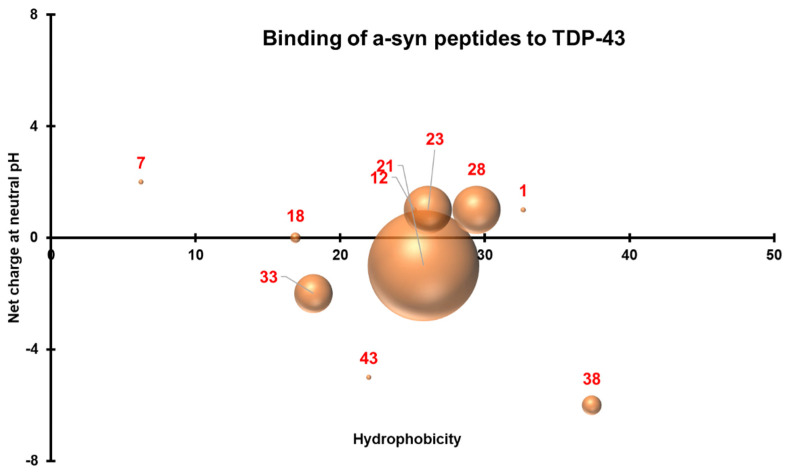
Three-dimensional scatterplot of α-synuclein peptides based on their hydrophobicity indices, net charge at pH 7.0, and their interactions with TDP-43. Peptides within the NAC region bound strongly to TDP-43.

To understand the properties that allowed the peptides to bind to their protein counterparts, a 3-D scatterplot of the hydrophobicity (*x*-axis), charge at neutral pH (*y*-axis), and the generated absorbances (*z*-axis) of the proteins that bound to the peptides was generated for TDP-43 (Figure 10) and α-synuclein (Figure 11). All values were calculated using an online Peptide Analyzing Tool (ThermoFisher). TDP-43 peptides that produced significant reactions by binding to full length α-synuclein have neutral charges (pH 7.0) and hydrophobicity indices that fall within the optimal region of 20–40 (Figure 10). On the other hand, non-overlapping peptides that cover the entire sequence of α-synuclein were selected and plotted similarly and these also had neutral charges (pH 7.0) and hydrophobicity indices within 20–30 (Figure 11). The similar properties of these peptides can be observed when the two graphs were overlapped. Neutral charges and hydrophobic values ranging from 20–40 were optimal for interaction to occur.

### 3.5. Conventional ELISA Did Not Show Any Interaction between the Full-Length Proteins

Applying the same microplate-based approach to investigate the interaction between the two full length monomeric proteins did not produce any significant signal relative to the background. The signals peaked at 2 ng/mL when the second protein was applied but the signals decreased when the concentrations were increased to 10 and 50 ng/mL. The luminescence values produced were very low and considered negligible (Figure S2). This may have resulted from the inhibition of the secondary structure of aa 310–329 in TDP-43, which contained a portion of helical domain. This helical domain and its proximity to the high affinity RRM domains may produce structural conformations in the solution that prevent this sequence from binding to full length α-synuclein. Additionally, binding may have been interrupted due to the steric hindrance from neighboring domains in the N-terminus of TDP-43. The domains on their own may have enough hydrogen, electrostatic and van der Waals forces to induce an interaction, but neighboring domains in the full-length sequence may have had an inhibiting influence on this interaction. C-terminal fragments (CTFs) are not an uncommon pathology of TDP-43 proteinopathy [26,27,28], and aa 310–329, particularly aa 311–314, may be influential in comorbid synucleinopathies. NMR analysis by Dhakal et al. showed that the TDP-43 LCD likely interacts with the N and C-termini of α-synuclein, evidenced by greater peak shifts in these regions [25]. However, these shifts were muted by the self-aggregation of TDP-43 LCD following incubation with α-synuclein [25]. Still, it is worth noting that only the LCD of TDP-43 was incorporated and without the interference of N-terminal domains.

### 3.6. In Silico Prediction of Protein Binding Affinity

Using ISLAND, the binding affinity and the k*_d_* between full-length TDP-43 and full-length α-synuclein were calculated to be −10.83 kcal/mol and 1.13 × 10^−8^ respectively. ISLAND incorporates machine learning to calculate ΔG and k*_d_* based on amino acid sequences. K*_d_*, in this case, was significantly low, thus the dissociation between the two proteins is much less likely to take place. This conflicted with the results acquired from ELISA, therefore other approaches may be recommended like isothermal titration calorimetry (ITC) to confirm this interaction. Moreover, the affinity prediction of ISLAND is sequence-based and other factors outside this sequence may affect the calculated parameters. It was stated that affinity prediction based on the peptide sequence is not satisfactory, and development of effective and practical methods in this domain is still an open problem [29]. However, this can still be considered a useful tool to explore the effects of various structural mutations on binding affinities and dissociation constants of two proteins to expand on their comorbid deposition in certain diseases.

## 4. Conclusions

Research on the comorbid interactions of TDP-43 with other pathological biomarkers is growing, and epitope mapping techniques are particularly useful in further characterizing interactions in these comorbidities. Here, a microplate-based immunoassay was used to determine interaction sites between TDP-43 and α-synuclein. A significantly high concentration of full-length α-synuclein was found to bind with aa 310–329 of the LCD in TDP-43. Using overlapping peptides that cover this region, MNFG residues (aa 311–324) were common to peptides that resulted in a high signal of bound α-synuclein. Thus, not only do TDP-43 CTFs initiate the formation of cytoplasmic inclusions by aggregating amongst themselves, but they can potentially create hybrid TDP-43/α-synuclein fibrils by interacting indiscriminately with all α-synuclein species. In contrast, a significant amount of full-length TDP-43 was found to bind with the NAC region of α-synuclein. Most importantly, co-incubation of human neuroblastoma SH-SY5Y cells with heterotypic fibrils composed of TDP-43 LCD and α-synuclein showed increased cellular toxicity when compared to SH-SY5Y cells incubated with either homotypic TDP-43 LCD or α-synuclein fibrils, reinforcing the pathological significance of this interaction [25].

Though other approaches such as NMR spectroscopy can better resolve these interactions, the self-aggregation of some regions can mute chemical peak shifts that indicate interaction sites. Separating the proteins as shorter peptides and testing binding for each peptide separately circumvents this problem. However, this approach can be limited when the actual site of interaction happens to be fragmented between multiple peptides and overlapping peptides in the suspected region must be designed and synthesized to resolve the interaction site. The length of each peptide also has to be estimated well so it can contain an optimal number of residues for binding to be observable. Finally, it is worth noting that the results generated herein employed recombinant proteins, thus, it may be necessary to confirm their physiological relevance via cell or animal models in future studies. Still, this approach is used because it is relatively convenient, economical, less tedious, and more rapid than other approaches.

## Data Availability

Data that support the findings of this study are available upon request.

## Supplementary Materials

The following are available online at https://www.mdpi.com/article/10.3390/jcm11030573/s1, Figure S1: Normalized bound α-synuclein to overlapping peptides of TDP-43, Figure S2: Conventional ELISA of serially diluted recombinant α-synuclein bound to fixed concentrations of TDP-43, and vice versa.
