# Supplementary material for: A Microplate-Based Approach to Map Interactions between TDP-43 and α-Synuclein
Source: J Clin Med. 2022 Jan 24;11(3):573. doi: 10.3390/jcm11030573 (PMC8836581; doi:10.3390/jcm11030573)
Supplement: Supplementary file 1 [file jcm-11-00573-s001.zip › jcm-1550222-supplementary.pdf]

Supplementary Materials

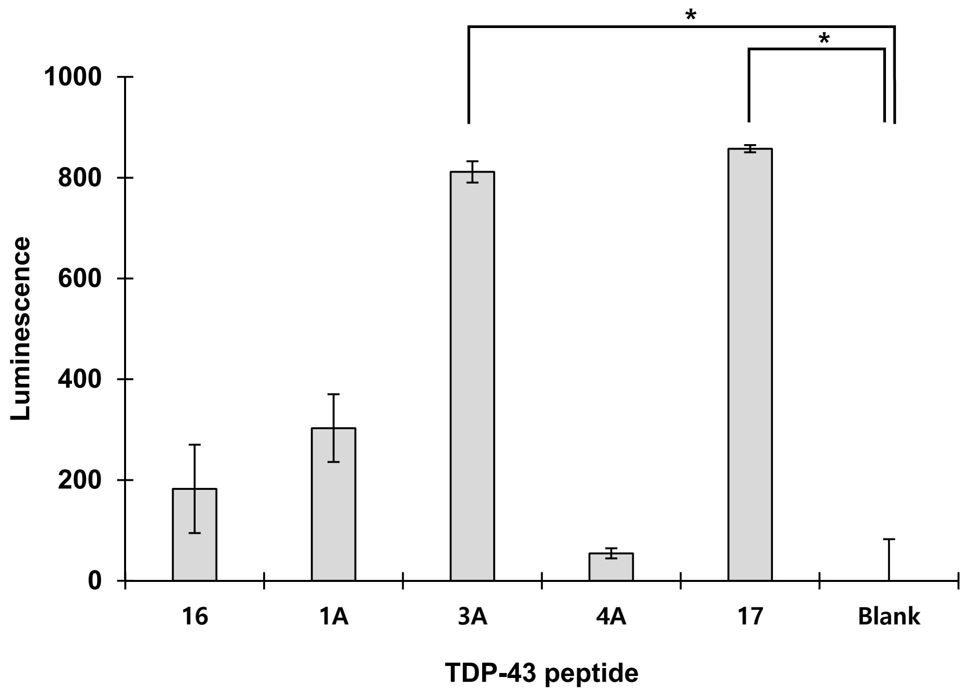

Figure S1. Normalized bound α-synuclein to overlapping peptides of TDP-43 (overlapping peptides are indicated with A). Data are shown as mean ± SD of duplicate measurement. Significant luminescence was observed for bound α-synuclein at peptides 1A and 3A when compared to the background ($p<0.05$).

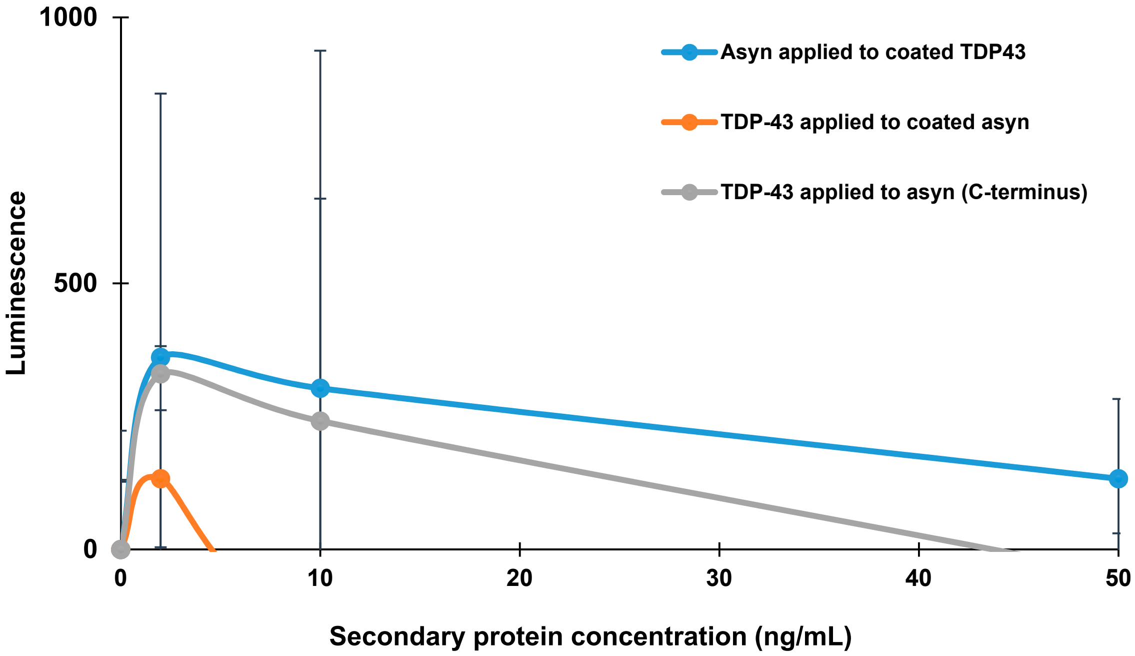

Figure S2. Conventional ELISA of serially diluted secondary protein bound to fixed concentrations of primary protein. Legends indicate the order in which the proteins were applied.
